# Long-Term Effect of Thinning on Radial Growth and Intrinsic Water Use Efficiency of a *Pinus koraiensis* Plantation on MountGari

**DOI:** 10.3390/plants15162471

**Published:** 2026-08-14

**Authors:** Kiwoong Lee, Soon Jin Yun, Minsu Kim, A Reum Kim

**Affiliations:** Division of Forest Ecology, National Institute of Forest Science, Seoul 02445, Republic of Korea; woongs0718@gmail.com (K.L.); lucksal7@naver.com (S.J.Y.); cealuver0492@gmail.com (M.K.)

**Keywords:** thinning intensity, drought resilience, intrinsic water use efficiency, tree-ring width, basal area increment

## Abstract

This study investigated the effects of thinning intensity on tree radial growth and physiological responses to drought in an approximately 43-year-old *Pinus koraiensis* plantation. To analyze tree-ring width, a total of sixty wood cores (5 mm) were collected from three thinning treatments applied in 2007: control (Con), light thinning (LT), and heavy thinning (HT). Drought vulnerability indices were examined across three distinct drought periods (2000–2001, 2007, and 2014–2016), and intrinsic water use efficiency (WUE*_i_*) was estimated from stable carbon isotope analysis (Con vs. HT). Thinning increased basal area increment (BAI) in both the LT and HT groups, with the strongest and most persistent response observed in the HT group. During the 2007 drought, trees in thinned plots, particularly those in the HT group, showed higher resistance and resilience than trees in the Con group. The increased indices during the 2000 and 2007 drought periods predominantly reflected immediate thinning-induced growth release. WUE*_i_* in the HT group increased relative to the Con group during both the 2007 and 2014–2016 drought periods; however, the underlying physiological mechanisms differed, with enhanced net photosynthetic capacity immediately after thinning in 2007 and a likely consistent reduction in stomatal conductance during the 2014–2016 drought. Although constrained by a non-replicated stand design, this site-specific long-term case study provides valuable insights into the multi-decadal growth and physiological trajectories of conifer plantations responding to climate stress.

## 1. Introduction

It is well documented that climate change is increasing the frequency and severity of drought events worldwide [1], which is expected to accelerate tree mortality, limit tree growth, and damage forest ecosystems [2]. Previous studies have found that drought stress causes higher tree mortality rates [3,4,5] and forest dieback [6,7,8], reduces net primary productivity and radial growth [9,10], and contributes to forest decline [11]. In particular, intensifying drought can induce hydraulic failure and carbon starvation in trees, severely impairing their physiological mechanisms [12,13].

Thinning is a major forest silvicultural practice that can improve tree growth, forest health, and stand water balance [14,15]. By reducing competition for water, light, and nutrients, thinning has also been reported to increase the resistance and resilience of remaining trees under drought stress [16,17,18]. To quantitatively evaluate tree responses to extreme climatic events, indices of drought vulnerability, such as resistance (*R*_t_), recovery (*R*_c_), and resilience (*R*_s_), have been widely used [19]. These indices allow for a detailed assessment of a tree’s capacity to withstand drought stress and its subsequent potential to return to pre-drought growth levels.

In South Korea, coniferous forests account for 37% of the total forest area (6,298,134 ha), and approximately 75% of this coniferous forest area is dominated by pine species. *Pinus koraiensis* forests account for 6.6% (151,579 ha) of coniferous forests [20]. *P. koraiensis* is one of the most important tree species both ecologically and economically, distributed across northeastern China, Korea, Russia, and Japan [21]. It provides high-value wood products and edible nuts [22]. Because of its shade tolerance, this species can survive under relatively low-light conditions, and its physiological traits allow it to become dominant in the climax successional stage [23]. As a high-value species, *P. koraiensis* in Korea has been investigated in various fields, including biomass and carbon storage [24,25], ecophysiology [26], nutrient cycling [27,28], and trade-offs between ecosystem services [29]. Nevertheless, *P. koraiensis* is known to be highly sensitive to rising temperatures and spring droughts, raising concerns about its long-term sustainability under future climate scenarios [30]. Despite this vulnerability, the long-term effects of thinning on tree growth and drought vulnerability remain unclear.

Physiologically, intrinsic water use efficiency (WUE*_i_*) is defined as the ratio of net photosynthetic rate (*A*) to stomatal conductance (*g*_s_). Thinning can alter WUE*_i_* through two distinct pathways: either by enhancing (*A*) due to increased light and nutrient availability, or by increasing *g*_s_ as a result of relieved soil water competition. Understanding these physiological mechanisms is crucial for interpreting the isotopic signals in tree rings.

Furthermore, understanding these long-term physiological and growth responses to thinning under changing climatic conditions provides crucial insights for developing adaptive forest management strategies.

In this study, we examined tree basal area increment (BAI), drought vulnerability indices (*R*_t_, *R*_c_, and *R*_s_), and intrinsic water use efficiency (WUE*_i_*) derived from carbon isotope composition (*δ*^13^C) under thinning and drought conditions. The objectives of this study were to: (1) investigate how thinning affects tree growth and drought vulnerability, (2) assess the response of WUE*_i_* to drought and thinning, and (3) analyze the relationship between tree-ring width and climatic variables.

## 2. Results

### 2.1. Temporal Changes in Basal Area Increment

**Figure 1:**
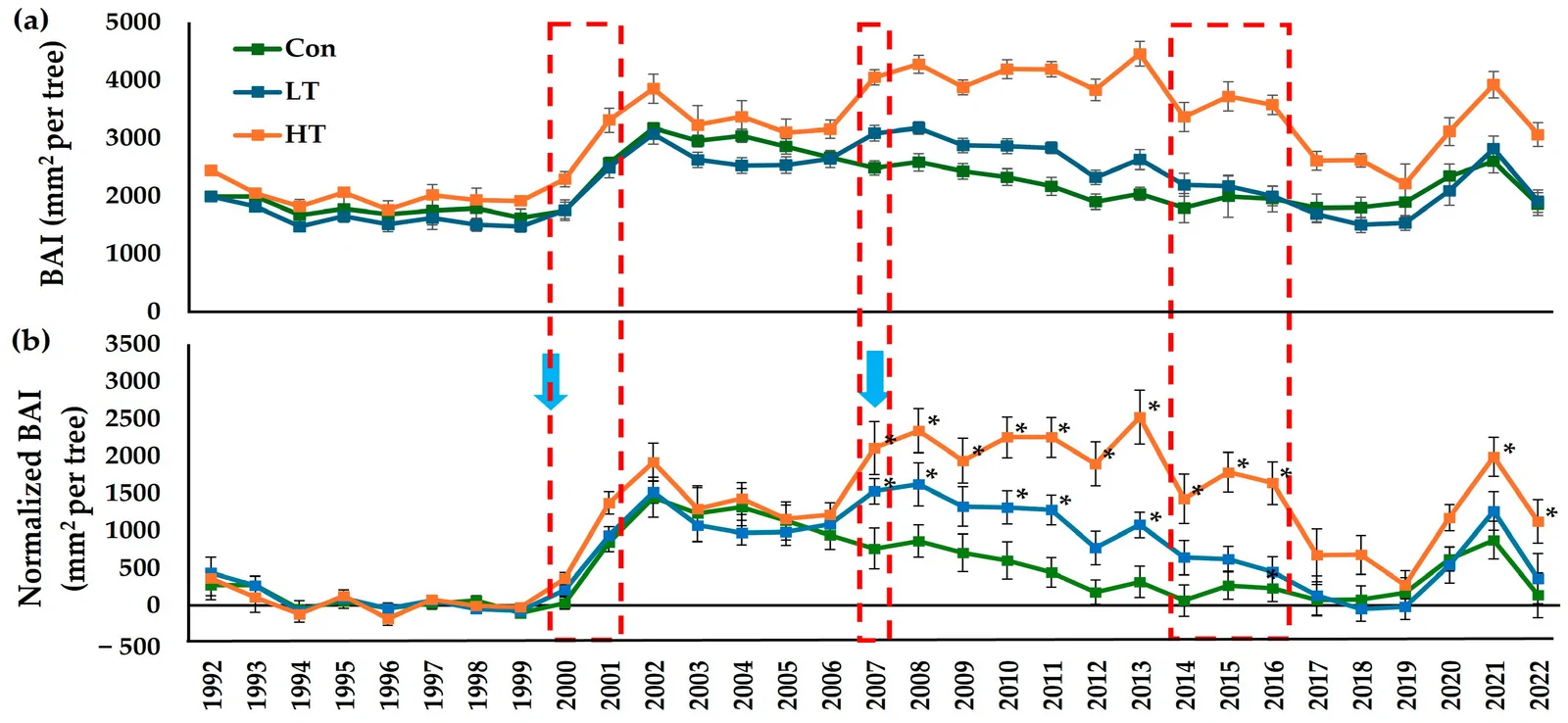
Changes in basal area increment (BAI) (**a**) and normalized BAI (**b**) across thinning intensities. The blue arrow indicates the timing of thinning (2000 and 2007), and the red dashed line represents the drought periods (2000–2001, 2007, and 2014–2016). Values were normalized by subtracting each tree’s pretreatment mean (1996–1999) from the values for each year in the tree’s time series. Asterisks (*) indicate significant differences from control values in a given year. (α = 0.05). Error bars are the standard error of the mean.

The linear mixed-effects model revealed significant main effects of thinning treatment (*F* = 9.02, *p* < 0.001), year (*F* = 18.45, *p* < 0.001), and their interaction (*F* = 2.14, *p* < 0.001) on normalized BAI (Table S1). To directly evaluate the post-thinning trajectory and eliminate baseline growth variations among individual trees, BAI values were normalized by subtracting each tree’s pretreatment mean (1996–1999) from its annual values. Consequently, the normalized values on the *y*-axis represent absolute deviations in growth relative to the pre-treatment baseline, where zero corresponds to the pretreatment average. The pre-commercial thinning conducted in 2000 increased basal area increment (BAI) across all treatments, with BAI reaching a peak in 2002, the second year after thinning. During the five years before thinning, the mean BAI was 1736.7 mm^2^. Following thinning, the mean BAI across treatments increased by 61.2% (1063.8 mm^2^) in 2001 and by 93.7% (1627.7 mm^2^) in 2002, relative to the pretreatment mean (Figure 1). Following the 2007 thinning, BAI showed distinct trajectories depending on thinning intensity, maintaining significant differences from the control (Con) group (*p* < 0.05), although responses varied by thinning intensity. In the heavy thinning (HT) group, BAI increased sharply in 2007 and remained elevated until 2013, before declining markedly in 2014 during a severe drought year. Despite this drought-related decline, a significant difference between the HT and Con groups was maintained from 2007 through 2022 (*p* < 0.05). In the light thinning (LT) group, BAI increased slightly following the 2007 thinning and subsequently showed a gradual downward trajectory through 2019. Although significant differences between the LT and Con groups were sporadically observed (e.g., in 2011 and 2015, *p* < 0.05), the LT group generally exhibited higher mean BAI values than the Con group until 2016, aligning with the extended years (such as 2007, 2008, 2010, 2011, and 2013) where positive tendencies emerged. In contrast, BAI in the Con group declined from 2007 to 2014 and then increased slightly from 2015 to 2021.

### 2.2. Radial Growth and Climate Relation

The response of radial growth to climatic variables varied with thinning intensity. Specifically, the ring-width indices (RWIs) in the Con group were negatively correlated with precipitation in the previous July, and with the 6-month Standardized Precipitation Evapotranspiration Index (SPEI-6) in the previous July and August. In contrast, RWI in the LT group increased in response to the mean temperature (*T*_mean_) in February, precipitation (*P*) in January, and SPEI from April to July of the current year. In the HT group, *P* was positively correlated with RWI in January of the current year. Moreover, RWI in the HT group was positively influenced by *T*_mean_ in February and by SPEI in May and June of the current year (Table 1).

### 2.3. Drought Vulnerability Indices

**Figure 2:**
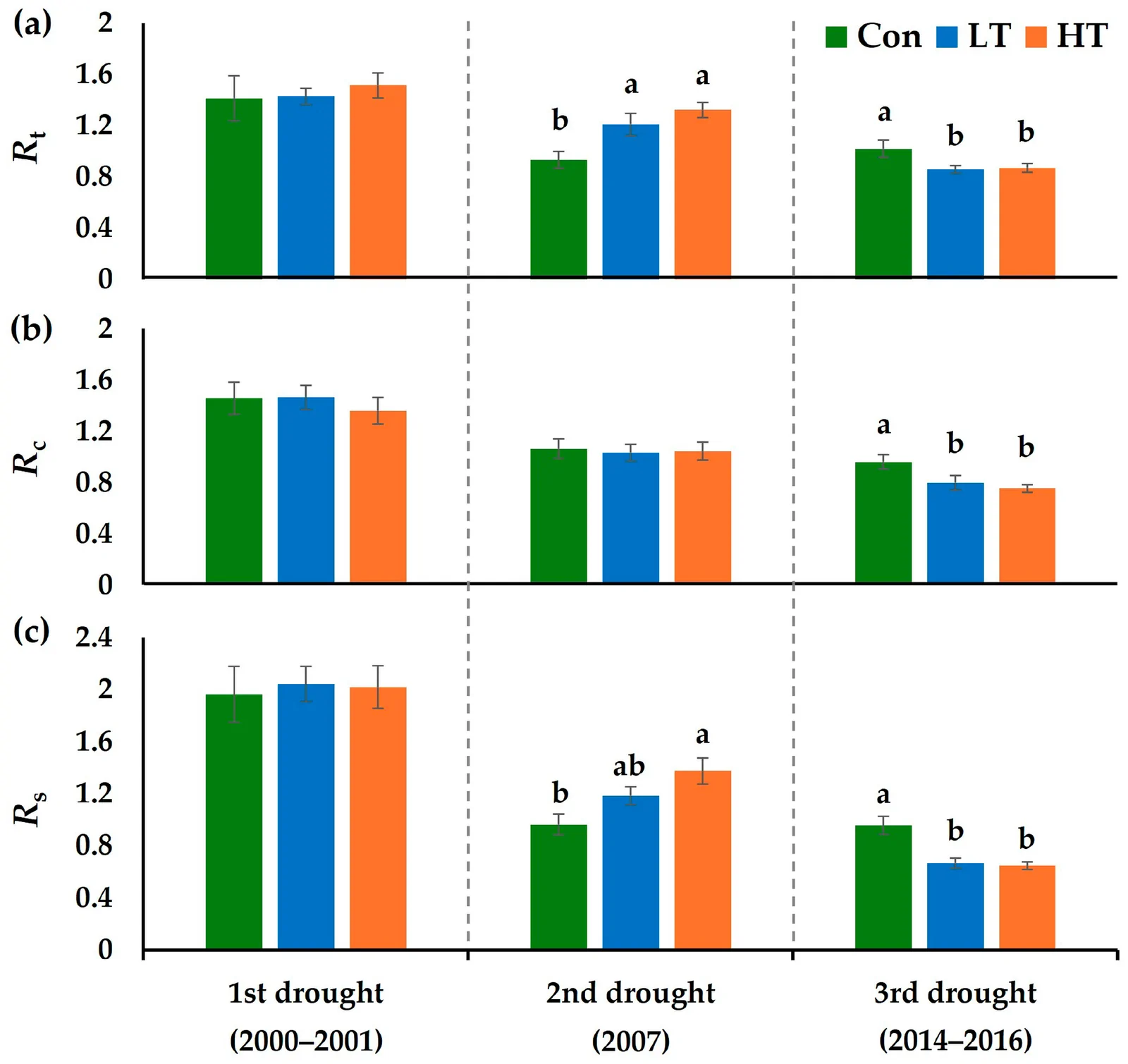
Resistance (**a**), recovery (**b**), and resilience (**c**) based on ring-width indices during three drought periods: 2000–2001, 2007, and 2014–2016. Different lowercase letters indicate significant differences among groups (*p* < 0.05). Error bars represent the standard error of the mean. Parametric comparisons were evaluated using one-way ANOVA followed by Tukey’s HSD post hoc test, whereas non-parametric comparisons (*R*_t_, *R*_c_ and *R*_s_ in 2000; *R*_t_ and *R*_c_ in 2007; *R*_c_ in 2014–2016) were evaluated using the Kruskal–Wallis test followed by Dunn’s post-hoc test.

Resistance (*R*_t_), recovery (*R*_c_), and resilience (*R*_s_) varied with thinning intensity across the three drought periods (Figure 2). Thinning had a great influence on the indices during the first drought period (2000–2001), with *R*_t_, *R*_c_, and *R*_s_ consistently exceeding 1, ranging from 1.4 to 2.0. During the second drought event, thinning intensity also had a significant effect on the indices. The LT and HT groups showed higher *R*_t_ values than the Con group, with their *R*_t_ exceeding 1 in both thinned groups (*p* < 0.05). Although *R*_s_ values in the LT and HT groups were greater than 1, only the HT group differed significantly from the Con group (*p* < 0.05). In contrast, *R*_c_ did not differ significantly among treatments, and the values consistently exceeded 1. During the third drought period (2014–2016), in contrast, all three indices in the Con group were significantly higher than those in the LT and HT groups (*p* < 0.05), with values close to 1.

### 2.4. Changes in Carbon Isotope Discrimination and Intrinsic Water Use Efficiency

**Figure 3:**
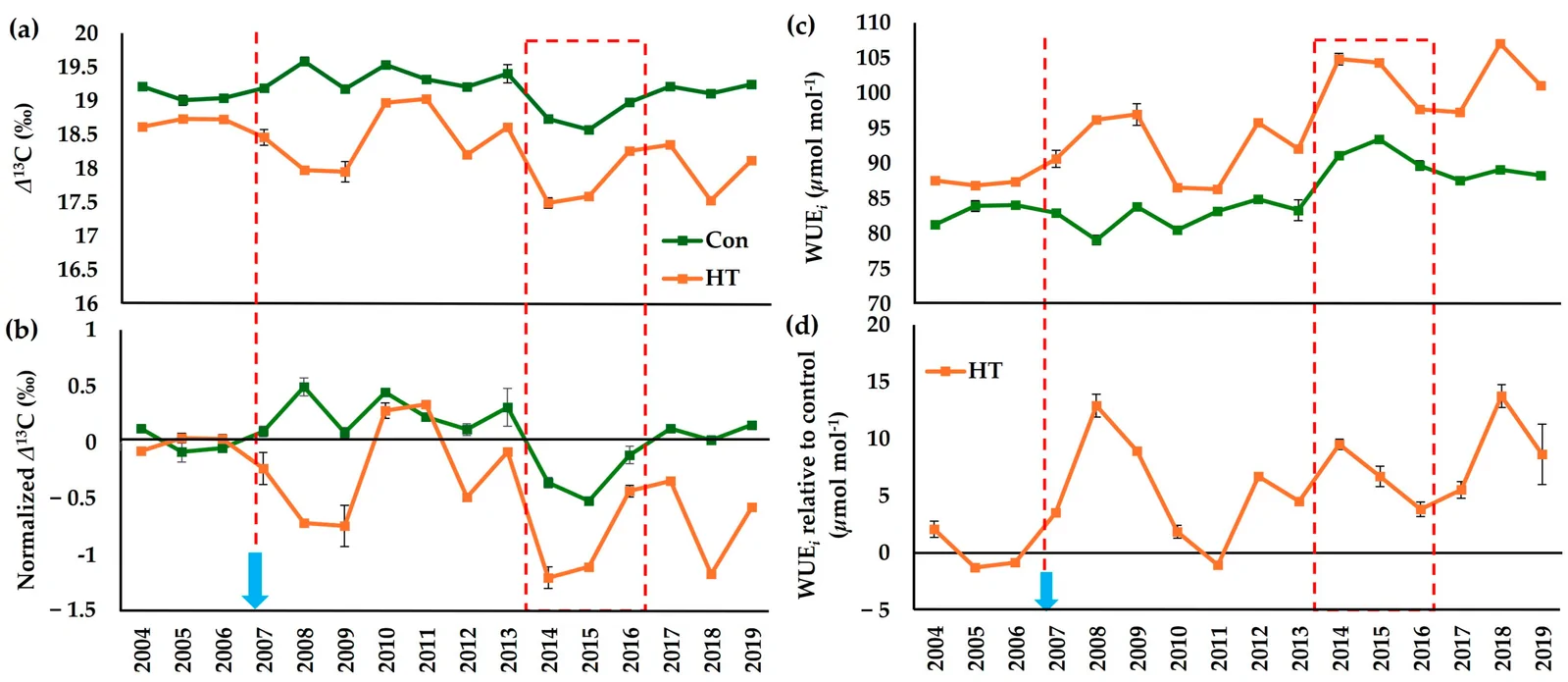
Inter-annual variations in physiological responses under thinning treatments. (**a**) Carbon isotope discrimination, (**b**) normalized carbon isotope discrimination, (**c**) intrinsic water use efficiency, and (**d**) normalized intrinsic water use efficiency. The blue arrow indicates the timing of thinning (2007), and the red dashed line represents the drought periods (2007 and 2014–2016). Values were normalized by subtracting each tree’s pretreatment mean (2004–2006) from the annual values in each year in the tree’s time series.

Carbon isotope discrimination (Δ^13^C) evaluated for the Con and HT groups varied from 17.3‰ to 20.1‰ across trees, treatments, and years (Figure 3). During the three years prior to thinning (2004–2006), the mean Δ^13^C was 18.7‰ across all treatments. In the HT group, Δ^13^C decreased by 0.7‰ from 2007 to 2009 relative to the pretreatment mean, reaching 17.9‰, which clearly reflects a post-thinning physiological shift (Figure 3a). This decrease in Δ^13^C corresponded to an increase in WUE*_i_* of approximately 10 μmol mol^−1^ in the HT group (Figure 3b). During the drought period from 2014 to 2016, a pronounced decrease in Δ^13^C was also observed in the HT group (Figure 3a,c), with values decreasing by 1.1‰ relative to the pretreatment mean. This reduction corresponded to an increase in WUE*_i_* of approximately 20 μmol mol^−1^ (Figure 3c).

## 3. Discussion

We investigated the effects of thinning on tree radial growth under drought conditions. To better understand these growth patterns, we also examined physiological responses and analyzed the relationships between climatic variables and radial growth.

### 3.1. The Effect of Thinning on Tree Growth

Our results showed that thinning applied in 2000 and 2007 was associated with increased tree radial growth, suggesting that thinning reduced competition and enabled residual trees to access more light, nutrients, and water resources [31,32]. This finding is consistent with findings from previous studies on thinning experiments in *P. koraiensi* forests [29,33]. Interestingly, the two thinned groups exhibited different trends after the 2007 thinning. Before the 2007 thinning, BAI was declining across all three groups, suggesting that the effect of the 2000 thinning was waning. However, the HT group showed a pronounced and sustained increase in BAI until 2022. This sustained growth response was likely attributable to repeated thinning combined with a substantial increase in resource availability after intensive density reduction [34,35]. In contrast, the LT group exhibited a more moderate change in BAI compared with the HT group, reflecting a more gradual response to the lighter density reduction.

Assessing thinning effects in long-term studies is essential for understanding the persistence of silvicultural impacts [36]. Our findings indicated that growth enhancements remained observable for 15 years after HT and for 9 years after LT. Although significant differences in the LT group were confined to specific years, this group consistently exhibited a positive growth trend relative to the Con group. Similar persistent thinning effects have been reported for other pine species, including sustained growth for 14–15 years in *P. nigra* [36], 20 years in *P. halepensis* after HT [37], and 12 years in *P. nigra* [38].

### 3.2. Relationships Between RWI and Climate

In the Con group, RWI showed negative correlations with moisture-related variables during the previous growing season. This suggests that the summer monsoon may have reduced the period of sufficient light availability for photosynthesis, or that reduced photosynthetic rates caused by low solar radiation and high relative humidity led to a deficit in the accumulation of non-structural carbohydrates (NSC) required for growth and survival [39]. In contrast, RWIs in both the LT and HT groups showed correlations with all variables. Specifically, the RWIs in both thinned groups showed a positive correlation with the current-year *T*_mean_, consistent with the results of [40,41]. This is likely due to canopy opening by thinning, which increases stand-level temperature and leads to an earlier onset of the growing season. Warm winters may reduce frost damage to plant tissues and facilitate normal metabolic activity in the following spring [40,42]. Furthermore, elevated winter temperatures promote earlier release from dormancy and earlier cambial activity, thereby accelerating cell expansion and extending the overall growth period [43,44]. Consequently, our results demonstrate that canopy opening through thinning modifies the forest microclimate and reshapes the climate–growth relationships of the remaining trees. In addition, the RWIs in both thinned groups were positively correlated with SPEI during the growing seasons. The LT group showed sensitivity to SPEI over a longer period than the HT group, suggesting that moderate canopy opening created favorable light and temperature conditions that allowed trees to use available water for growth over an extended period. In contrast, the extensive canopy opening in the HT group may have increased light exposure and soil temperature, thereby increasing evaporative demand; consequently, trees in the HT group appeared to concentrate growth in May and June before water availability became limiting.

### 3.3. The Effects of Thinning on Drought Vulnerability Indices

The first drought period (2000–2001) coincided with the thinning applied in 2000. Despite the drought, all three indices across the three groups were greater than 1. This indicates that tree growth during the drought was higher than pre-drought growth, suggesting that the growth increase induced by thinning offset and exceeded any drought-related reduction in growth.

The second drought period (2007) also coincided with the thinning treatments. In both thinned groups, *R*_t_ and *R*_s_ were greater than 1, whereas *R*_c_ values were close to 1 across all three groups. *R*_c_ is calculated as the ratio of BAI_Post_ to BAI_D_; thus, a value at or below 1 indicates that post-drought growth did not exceed growth during the drought, suggesting a persistent drought effect. Although *R*_c_ was approximately 1 and did not differ significantly among the three groups, the ecological implications differed fundamentally between the thinned groups and the Con group. After thinning in 2007, BAI in the HT and LT groups increased by approximately 1600 mm^2^ and 600 mm^2^ in 2008, respectively, compared with the Con group. This indicates that the positive effect of thinning persisted beyond the drought period, allowing the remaining trees to maintain increasing growth rates. Conversely, in the Con group, the *R*_c_ value of approximately 1 indicates that tree growth remained stagnant at low levels even after the drought period.

In agreement with [38], this study highlights that relying solely on drought vulnerability indices without accounting for baseline growth can lead to an underestimation of the effect of thinning. During the third drought period (2014–2016), all three indices in the Con group were higher than those in the thinned groups. Although the Con group had higher index values, all values were close to 1 (*R*_t_ = 1.01, *R*_c_ = 0.96, and *R*_s_ = 0.96), whereas the indices in the thinned groups were less than 1. This result could lead to the misleading impression that thinning increases drought vulnerability; however, it actually reflects a mechanical artifact caused by baseline growth differences, a phenomenon also highlighted by Manrique-Alba et al. [38]. Before the third drought period, the BAI in both thinned groups was substantially higher than in the Con group. Consequently, the drought-induced reductions in BAI were more pronounced in the thinned groups than in the Con group, which likely explains the decline in their drought indices. In contrast, the BAI in the Con group showed negligible change from the pre-drought to the post-drought period, resulting in all three indices remaining close to 1. This suggests that the trees in the Con group were already under chronic stress induced by intense competition before the drought period.

### 3.4. The Effects of Thinning on Carbon Isotope Discrimination and Intrinsic Water Use Efficiency

Our results indicate that the physiological mechanisms driving the increase in WUE*_i_* differed between the two drought periods. During the first two years after thinning, Δ^13^C decreased while WUE*_i_* increased. This pattern suggests that the observed trends may reflect enhanced photosynthetic capacity rather than changes in *g*_s_, whether increased or stable [45,46,47]. Previous research has demonstrated that thinning opens the tree canopy, modifies the forest microclimate, and exposes leaves of remaining trees to higher light intensity, thereby increasing net photosynthesis (*A*). This results in higher *δ*^13^C, reflecting a decrease in Δ^13^C, and increased WUE*_i_* [48,49,50]. In contrast, after the first two years, WUE*_i_* decreased. This is likely due to increased water availability resulting from reduced competition after thinning. This subsequent decrease highlights that the increased WUE*_i_* during the first two years was primarily driven by enhanced photosynthetic capacity under higher light availability, rather than by changes in water status.

Our results are consistent with previous thinning studies that reported increased WUE*_i_* in *P. taeda* [45] and *P. massoniana* [49] in mesic areas after thinning. However, our results differ from previous findings for *P. halepensis* [37] and *P. ponderosa* [51] in drier, semiarid areas, which exhibited decreased WUE*_i_* after thinning. These contrasting WUE*_i_* trends may depend on environmental conditions. Sites dominated by *P. halepensis* and *P. ponderosa* are water-limited or drought-prone; therefore, thinning may primarily increase stomatal conductance through improved water availability rather than enhance photosynthetic capacity through increased light availability.

During the drought period from 2014 to 2016, WUE*_i_* also increased, consistent with expectations under drought stress. This increase in WUE*_i_* is likely consistent with a reduction in *g*_s_. Under drought conditions, plants reduce stomatal conductance to prevent water loss, which increases WUE*_i_* [52,53,54]. This result is in agreement with previous studies [55,56,57]. Finally, while using whole ring tissue rather than separating earlywood and latewood could potentially introduce carry-over effects from previous year reserves, previous studies on pine species have demonstrated that whole-ring *δ*^13^C highly correlates with latewood *δ*^13^C and reliably captures inter-annual isotopic trends [58,59]. Nevertheless, incorporating intra-annual isotopic analysis across all thinning intensities will help evaluate fine-scale physiological responses.

A methodological consideration of this study is that the thinning treatments were established as single contiguous stands rather than spatially replicated blocks. According to rigorous experimental design criteria [60], observations from multiple plots within a single treated stand operate as subsampling units rather than true independent replicates, limiting the scope of statistical spatial inference. Consequently, our findings should be interpreted as a long-term, site-specific case study reflecting operational-scale stand dynamics at Mt. Gari, rather than universally generalizable causal effects. Nonetheless, the clear temporal alignment of physiological and growth shifts following the 2007 intervention provides robust empirical insights into the long-term trajectories of *P. koraiensis* subjected to varying thinning intensities.

## 4. Materials and Methods

### 4.1. Study Site

**Figure 4:**
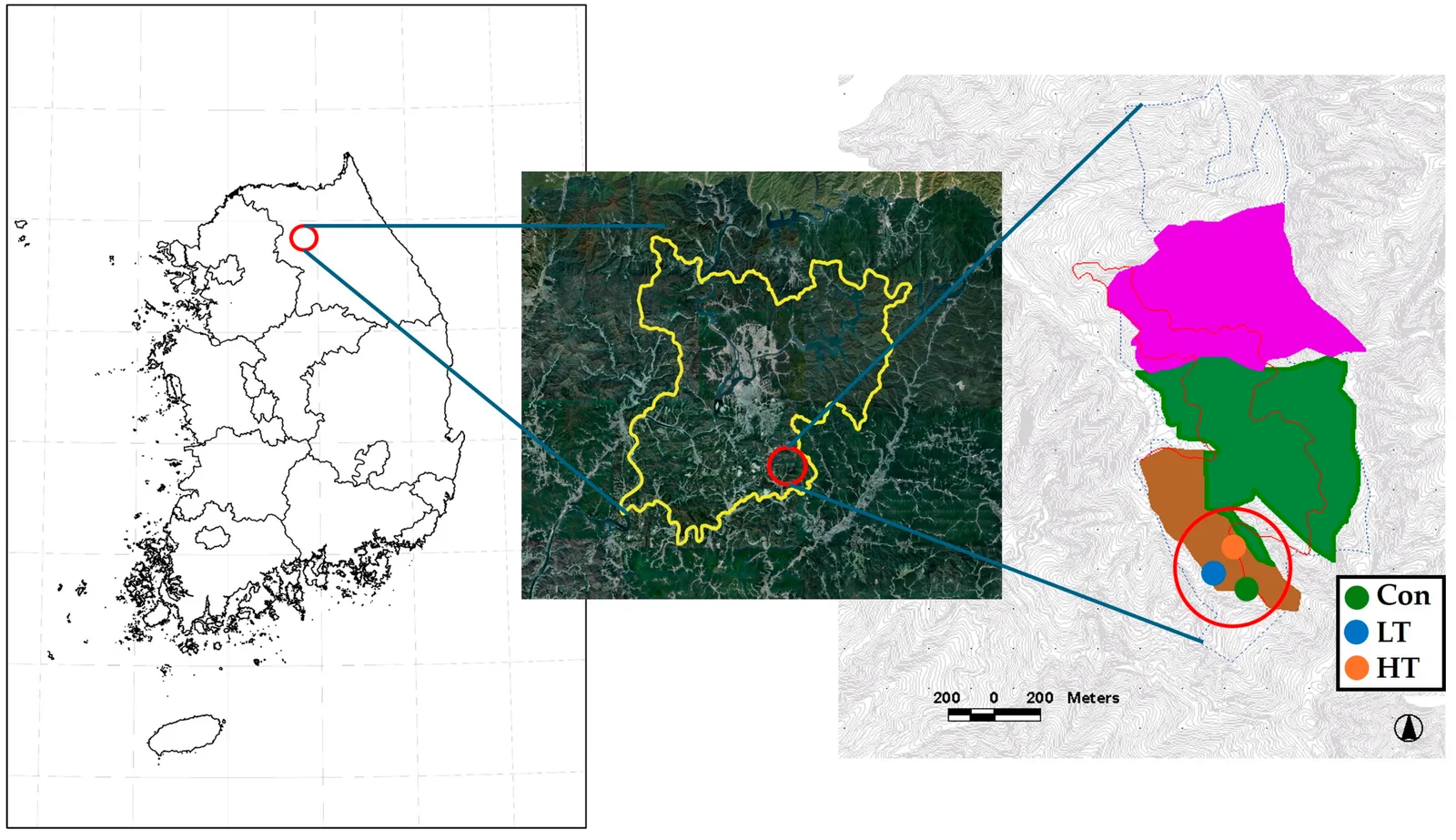
Location of the study site on Mt. Gari. Green, blue, and orange circles indicate areas with control, light thinning, and heavy thinning treatments, respectively.

This study was conducted in a Korean pine plantation at Mt. Gari in Chuncheon, Gangwon Province, South Korea (37°52′49.12″ N, 127°52′30.11″ E) (Figure 4). The total plantation area was 18 ha, planted in the early 1980s. From 2000 to 2020, monthly mean air temperature ranged from −4.6 °C to 24.6 °C and mean annual air temperature averaged 11.1 °C (± 0.12 °C); mean annual precipitation was 1347.3 mm (± 70.8 mm) [29]. The study plots were established in 2006 at elevations of 430 to 500 m above sea level, with slopes ranging from 10° to 26° and a westward aspect. Before plot establishment in 2006, a pre-commercial thinning (~25% reduction based on stand density [SD]) was applied across the entire plantation area in 2000. Four 20 m × 20 m plots were established for each treatment. In 2005, before experimental thinning, the mean diameter at breast height (DBH) at 1.2 m and the SD in 2005 were 22.5 cm and 573.3 trees ha^−1^, respectively. Experimental thinning was implemented in 2007 across large-scale, contiguous forest stands measuring 2.2 ha for the control (Con, unthinned), 7 ha for light thinning (LT, ~35% reduction based on SD), and 5 ha for heavy thinning (HT, ~65% reduction based on SD). Within each of these treatment stands, four 20 m × 20 m plots were established not as isolated experimental units, but as sub-sampling frameworks to standardize structural measurements and account for micro-environmental variation. Site characteristics 15 years after thinning are presented in Table 2.

### 4.2. Wood Core Sampling

Forest inventory was carried out in March 2023. The DBH and height (H) were measured using diameter tapes and a rangefinder (Vertex 5; Haglöf, Långsele, Sweden), respectively. In each treatment, we selected 18–20 healthy, dominant, or co-dominant sample trees with DBH values similar to the stand mean. We used two types of increment borers: (1) 5.15 mm cores for ring-width analysis, and (2) 12 mm cores for carbon isotope analysis. To minimize bias associated with reaction wood, the cores were collected at breast height (1.2 m) in directions perpendicular to the slope direction [61]. For tree-ring width analysis, twenty 5.15 mm cores were extracted per treatment, resulting in a total of sixty 5.15 mm cores. In addition, ten 12 mm cores per treatment were collected for carbon isotope analysis.

### 4.3. TreeRing Width Measurement and Chronology

**Figure 5:**
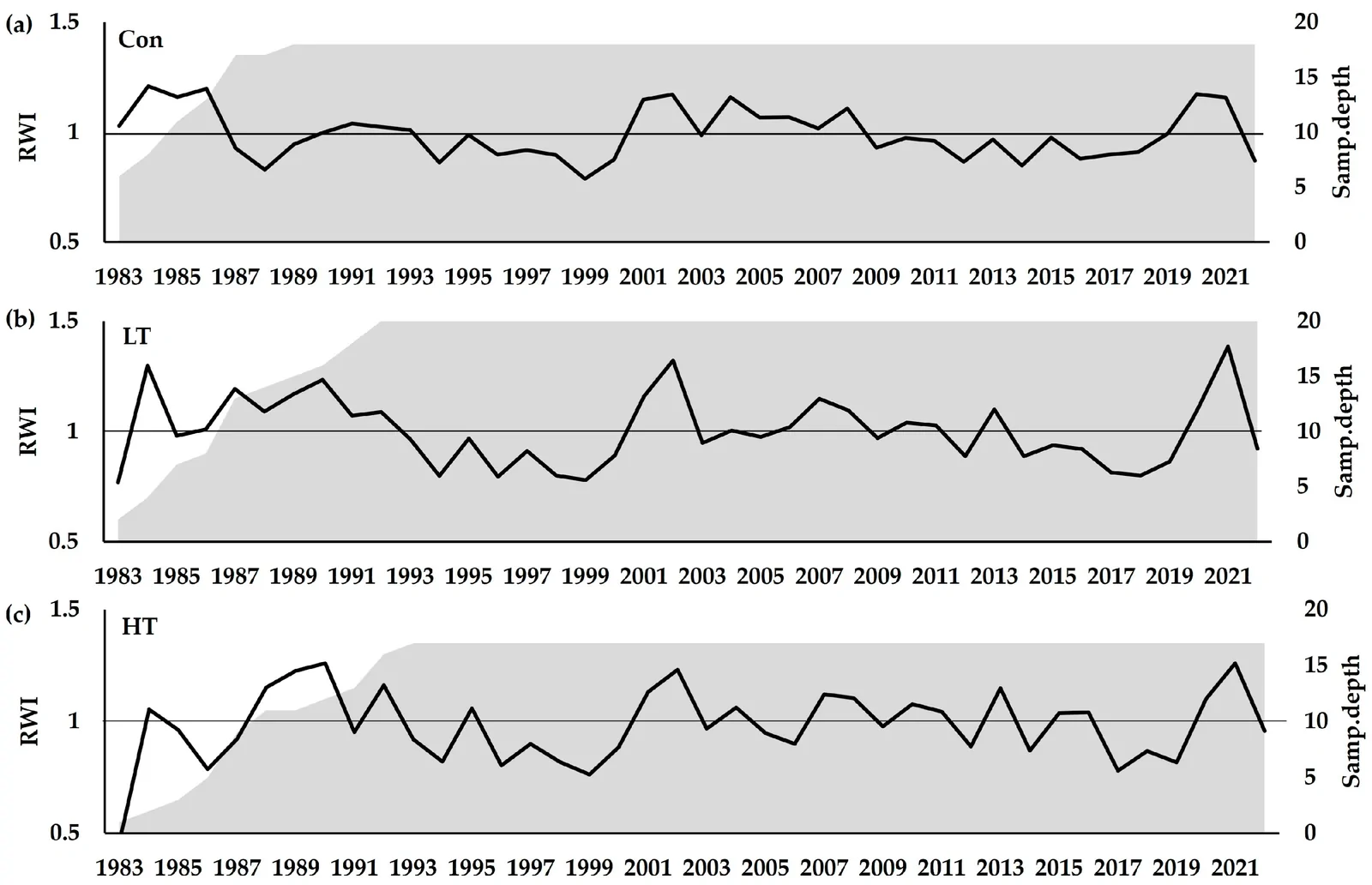
Standardized chronologies of tree-ring width index (RWI) under thinning treatments: (**a**) control, (**b**) light thinning, and (**c**) heavy thinning. Solid black lines represent the RWI, and the gray shaded areas indicate the sample depth (number of cores) used for each chronology.

For ring-width measurements, the 5.15 mm diameter cores were dried, mounted on wooden supports, and sanded with progressively finer sandpaper (200–600 grit) until the ring boundaries were clearly visible. The cores were then scanned at 2400 dpi using an Epson V370 scanner (Epson Corp., Nagano, Japan). Ring width was measured with a precision of 0.01 mm, and cross-dating was performed on whole cores using CDendro 9.8.1 software. We standardized the ring-width series to build a chronology for each treatment using the dplR package in R, with a spline curve with a 50% frequency response at a wavelength of two-thirds of the series length [62] (Figure 5).

Additionally, the following dendrochronological statistics were calculated to characterize the chronologies: mean sensitivity (MS), mean inter-series correlation (R_bar_), expressed population signal (EPS), subsample signal strength (SSS), autocorrelation (AR_1), and signal-to-noise ratio (SNR) (Table 3). For data analysis, we used years with SSS values above 0.85, which corresponded to the period from 1993 to 2022.

To calculate basal area increment (BAI), annual diameters were reconstructed from DBH measured in 2023 and annual ring widths and then converted to BAI using the formula below:

BAI_t_ = π × (*R*^2^_t_ − *R*^2^_t−1_) (1)

where BAI_t_ is the annual basal area increment in a particular year t, and *R*_t_ and *R*_t−1_ denote the stem radius in the current year and the previous year, respectively, assuming that stem growth approximates the area of a circle.

Drought periods were identified based on a combination of meteorological drought severity and physiological growth responses (OR logic). Specifically, years were designated as drought periods if they met either of the following criteria: (1) severe drought (SPEI-6 ≤ −1.5 [63]), or (2) a growth reduction characterized by a BAI decline of ≥10% [64,65] relative to the preceding 3-year mean baseline [19]. We applied a short 3-year baseline because using a longer period causes thinning effects or other drought events to disturb climate signals [19,66].

Under these criteria, the 2000–2001 and 2014–2016 periods were identified primarily by severe meteorological drought (SPEI-6 ≤ −1.5), whereas the 2007 drought period was identified based on significant growth suppression (BAI reduction ≥ 10%), despite SPEI-6 reaching −1.2 (moderate drought).

Monthly mean temperature and total precipitation data from 1981 to 2022 were obtained from the Korea Meteorological Administration (KMA; https://data.kma.go.kr, accessed on 3 March 2025) to calculate the 6-month Standardized Precipitation Evapotranspiration Index (SPEI-6). Potential evapotranspiration was estimated using the Thornthwaite method based on monthly mean temperature and site latitude, and SPEI-6 values were derived using the SPEI package in R software (version 4.1.2; R Core Team, Vienna, Austria). Finally, we calculated the drought vulnerability indices suggested by [19] for the three identified drought periods (2000–2001, 2007, and 2014–2016).

Three drought vulnerability indices proposed by [19] were calculated as follows:

(2) $$\mathrm{Resistance}\;(R_{t})=\frac{{\mathrm{BAI}}_{D}}{{\mathrm{BAI}}_{\mathrm{Pre}}}$$

(3) $$\mathrm{Recovery}\;(R_{c})=\frac{{\mathrm{BAI}}_{\mathrm{Post}}}{{\mathrm{BAI}}_{D}}$$

(4) $$\mathrm{Resilience}\;(R_{s})=\frac{{\mathrm{BAI}}_{\mathrm{Post}}}{{\mathrm{BAI}}_{\mathrm{Pre}}}$$

where BAI_D_ refers to the BAI during the drought period, while BAI_Pre_ and BAI_Post_ represent the mean BAI of the two-year periods preceding and following the drought, respectively.

### 4.4. Climate-Growth Relationships

The relationship between detrended ring width and climate variables—including monthly mean air temperature, precipitation, and SPEI-6—was analyzed. Correlation functions were computed from June of the previous year to September of the current year. This analysis was performed for the period from 1983 to 2022 using the treeclim package in R software.

### 4.5. Carbon Isotope and Intrinsic Water Use Efficiency

To assess the physiological mechanisms underlying thinning, stable carbon isotope analysis was specifically conducted on the Con and HT groups. Five trees per plot were selected from the Con and HT groups that were highly correlated with overall plot-level tree-ring chronology [37,38]. The 12 mm cores were dissected into whole rings using a razor blade. In this study, we used the entire ring instead of separating earlywood and latewood to obtain sufficient wood material. The dissected annual rings were milled and pooled by year. The powdered samples were delivered to the National Instrumentation Center for Environmental Management (NICEM) at Seoul National University for stable carbon isotope analysis using an isotope ratio mass spectrometer (IsoPrime-EA, Micromass, Wilmslow, UK).

Carbon composition (*δ*^13^C) and carbon isotope discrimination (Δ^13^C) were used to calculate intrinsic water use efficiency (WUE*_i_*). *δ*^13^C is expressed as follows:

(5) $$\delta^{13}C\;(‰)=[\frac{R_{\mathrm{sample}}}{R_{\mathrm{standard}}}-1]\times 1000$$

where *R* represents the ^13^C/^12^C ratio. The Δ^13^C can be expressed as based on the carbon isotope discrimination model:

(6) $$\delta^{13}C_{p}\;(‰)=\delta^{13}C_{a}-a-(b-a)\times \frac{c_{i}}{c_{a}}$$

where *δ*^13^C_a_ and *δ*^13^C_p_ denote the ^13^C/^12^C ratio in the ambient air and the plant material, respectively. *a* is the discrimination due to diffusion (4.4‰) and *b* is the discrimination due to carboxylation (27‰). Additionally, *c*_a_ and *c*_i_ denote the ambient and intercellular concentrations of CO_2_, respectively. *c*_a_ values were obtained from the NOAA database (https://gml.noaa.gov/data/data.php?category=Greenhouse%252BGases&parameter_name=Carbon%2BDioxide, accessed on 9 February 2026), and *δ*^13^C_a_ data were obtained from the Scripps CO_2_ Program (SIO) (https://scrippsco2.ucsd.edu/data/atmospheric-co2-data/sampling-station-records/mauna-loa-observatory-hawaii, accessed on 10 February 2026). The Δ^13^C can be expressed as follows:

(7) $$\Delta^{13}C_{p}\;(‰)=\frac{\left(\delta^{13}C_{a}-\delta^{13}C_{p}\right)}{\left(1+\delta^{13}C_{p}\right)}$$

(8) $$\Delta^{13}C_{p}\;(‰)=a+(b-a)\times \frac{c_{i}}{c_{a}}$$

(9) $${\mathrm{WUE}}_{i}\;({\mu \mathrm{mol}\;\mathrm{mol}}^{-1})=\frac{A}{g_{s}}=\frac{\left(c_{a}-c_{i}\right)}{1.6}$$

where WUE*_i_* is defined as the ratio of net carbon assimilation (*A*) to stomatal conductance (*g*_s_), and 1.6 is the ratio of the diffusivities of water and CO_2_ in the atmosphere.

### 4.6. Statistical Analysis

For the tree-level growth dynamics, chronological variations in normalized BAI (Figure 1) were evaluated using a linear mixed-effects model (LMM) via the nlme package in R. The model specified treatment (Group), year (treated as a categorical factor), and their two-way interaction as fixed effects, while individual tree identity was incorporated as a random intercept to account for the hierarchical data structure and repeated measurements on the same individuals over decades. To explicitly account for temporal autocorrelation among successive annual observations, a first-order autoregressive correlation structure (corAR1(form = ~ Year_num | Tree_ID)) was incorporated into the residuals. Individual trees were selected as the primary unit of analysis because thinning treatments were applied across large-scale, contiguous stand areas (2.2–7 ha) and tree-level growth dynamics reflect fine-scale local competition and micro-climatic variations rather than plot-level boundaries. Visual inspection of residual plots confirmed that the assumptions of normality and homoscedasticity were well satisfied without data transformation. To control for Type I error inflation during multiple year-by-year pairwise comparisons, post hoc pairwise treatment contrasts within each specific year were evaluated using estimated marginal means (emmeans package) with a Bonferroni adjustment. Model adequacy and goodness-of-fit were assessed using Akaike’s Information Criterion (AIC = 25,175.75), and the coefficients of determination were calculated via the MuMIn package: the marginal *R*^2^ ($R_{m}^{2}$, variance explained by fixed effects) was 0.376, and the conditional *R*^2^ ($R_{c}^{2}$, variance explained by both fixed and random effects) was 0.506 (Table S1). For the stable isotope datasets (Δ^13^C and WUE*_i_*), no LMM was applied due to the operational pooling of samples by year across replicates.

One-way ANOVA was performed to test for differences in resistance, recovery, and resilience among treatments. When the assumptions of normality or homoscedasticity were not met, the non-parametric Kruskal–Wallis test was used. Post hoc analyses were conducted using Tukey’s HSD test after ANOVA and Dunn’s test after the Kruskal–Wallis test. Specifically, non-parametric tests were applied to all three indices (*R*_t_, *R*_c_, and *R*_s_) in the 2000 drought period, *R*_t_ and *R*_c_ in the 2007 drought period, and *R*_c_ in the 2014–2016 drought period. All remaining indices were evaluated using parametric one-way ANOVA with Tukey’s HSD test. The relationship between climatic variables and tree-ring width was analyzed using bootstrapped Pearson’s correlation with the treeclim package in R. BAI, Δ^13^C, and WUE*_i_* values were normalized to account for pretreatment differences. BAI was normalized by subtracting each tree’s pretreatment mean for 1996–1999, whereas Δ^13^C and WUE*_i_* were normalized by subtracting the 2004–2006 pretreatment mean. For WUE*_i_*, treatment differences were estimated by subtracting normalized control values from normalized treated values [48]. All statistical analyses were performed using R software (version 4.5.3).

## 5. Conclusions

While thinning practices in 2000 and 2007 masked immediate drought impacts through growth release, the subsequent 2014–2016 drought confirms that thinning provides long-term forest resilience against climate stress in a 43-year-old *P. koraiensis* plantation. In particular, HT had a positive effect on BAI that persisted for up to 15 years. Δ^13^C analysis revealed the mechanisms underlying the increased WUE*_i_* in the HT group: WUE*_i_* increased immediately after thinning because of enhanced photosynthetic capacity driven by greater light availability, whereas the increase in WUE*_i_* during a drought event several years after thinning was governed by stomatal regulation to prevent water loss. These results demonstrate distinct physiological mechanisms over time. However, because this study used carbon isotopes alone, further research incorporating additional isotopes, such as *δ*^18^O, is needed to obtain a more comprehensive understanding of WUE*_i_* responses to thinning and drought. Overall, our findings provide a scientific basis for forest management decision-making under future climate change scenarios and may contribute to the development of forest management guidelines for enhancing the climate resilience of conifer plantations facing increasing drought risk.

## Figures and Tables

**Table 1:** Significant climate–radial growth relationships across thinning intensities from April of the previous year to September of the current year. Only significant correlations are shown (*p* < 0.05). Red and blue arrows denote negative and positive correlations, respectively. Lowercase month labels indicate months of the previous year, and uppercase labels indicate months of the current year. *T*_mean_ and *P* denote mean temperature and precipitation, respectively.

Climatic Variables	Month	Con	LT	HT
*T*_mean_	FEB	-	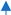 0.37	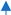 0.39
*P*	jul	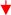 −0.30	-	-
*P*	JAN	-	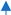 0.31	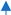 0.31
SPEI	jul	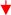 −0.32	-	-
SPEI	aug	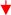 −0.27	-	-
SPEI	APR	-	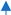 0.31	-
SPEI	MAY	-	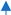 0.27	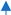 0.28
SPEI	JUN	-	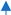 0.38	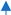 0.36
SPEI	JUL	-	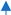 0.29	

**Table 2:** Characteristics of the study site in 2023. Con, LT, and HT indicate control, light thinning, and heavy thinning, respectively.

	Con	LT	HT
Elevation (m)	438	446	454
Aspect	SW	W	SW
Slope (°)	10	15	26
Mean DBH (cm)	33.2 (±1.07)	36.4 (±0.48)	42.1 (±0.96)
Basal area (m^2^ ha^−1^)	47.5 (±1.83)	39.9 (±2.13)	27.8 (±1.46)
Stand density (trees ha^−1^)	543.8 (±24.3)	387.5 (±28.6)	200.0 (±15.3)

**Table 3:** Descriptive statistics of site chronologies during the common period.

Treatment	Cores/Trees	DBH (cm)	AR_1	R_bar_	EPS	SSS	SNR	MS
Con	18/20	32.7 (±0.56)	0.64	0.500	0.947	0.93	27.079	0.255
LT	20/20	33.1 (±0.40)	0.61	0.575	0.964	0.97	23.888	0.275
HT	17/20	38.3 (±0.46)	0.49	0.534	0.951	0.95	32.009	0.251

## Data Availability

The data presented in this study are available on reasonable request from the corresponding author.

## Supplementary Materials

The following supporting information can be downloaded at: https://www.mdpi.com/article/10.3390/plants15162471/s1, Table S1: Results of the linear mixed-effects model examining the effects of thinning treatment, year, and their interaction on basal area increment (BAI).

## Abbreviations

The following abbreviations are used in this manuscript:

SPEI	Standardized Precipitation Evapotranspiration Index
R_bar_	Mean inter-series correlation
WUE*_i_*	intrinsic water use efficiency
RWI	Ring width index
BAI	Basal area increment
EPS	Expressed population signal
SSS	Subsample signal strength
SNR	Signal-to-noise ratio
HT	Heavy thinning
LT	Light thinning
MS	Mean sensitivity
