# Supplementary material for: Long-Term Effect of Thinning on Radial Growth and Intrinsic Water Use Efficiency of a Pinus koraiensis Plantation on MountGari
Source: Plants (Basel). 2026 Aug 14;15(16):2471. doi: 10.3390/plants15162471 (PMC13517011; doi:10.3390/plants15162471)
Supplement: Supplementary file 1 [file plants-15-02471-s001.zip › plants-4417047-supplementary.pdf]

Table S1. Results of the linear mixed-effects model examining the effects of thinning treatment, year, and their interaction on basal area increment (BAI)

Source / Parameter	*F*-value	numDF	denDF	*p*-value / Value
**Fixed Effect**				
(intercept)	117.54	1	1,508	< 0.0001
Treatment (Group)	9.02	2	52	< 0. 001
Year	18.45	29	1,508	< 0. 001
Treatment × Year	2.14	58	1,508	< 0. 001
**Random Effects**				
Tree variance (Sample_ID)				167,830.3
Residual Variance				633,943.2
**Model Fit Parameters**				
Akaike's Information Criterion (AIC)				25,175.75
Bayesian Information Criterion (BIC)				25,673.53
Log-Likelihood				-12,494.88
Marginal R^2 (R^2_m)				0.376
Conditional R^2 (R^2_c)				0.506

Note: Values are based on a linear mixed-effects model incorporating an AR(1) autocorrelation structure (φ = 0.507).
